# A Comparative Analysis of the Ecological Niches of *Erigeron canadensis* L. and *Erigeron annuus* (L.) Pers. in Invasive Areas and Their Native Habitats

**DOI:** 10.3390/life16101674

**Published:** 2026-10-07

**Authors:** Wansheng Liu, Jiale Liu, Mo Zhou, Liqiang Mu

**Affiliations:** 1 College of Forestry, Northeast Forestry University, Harbin 150040, China; liuwansheng1995@163.com (W.L.); ljiale1204@163.com (J.L.); 2 Horticultural Branch, Heilongjiang Academy of Agricultural Sciences, Harbin 150069, China; 13816949870@163.com

**Keywords:** biological invasions, *Erigeron canadensis* L., *Erigeron annuus* (L.) Pers., ecological niche, spatial niche overlap

## Abstract

Biological invasions are a major threat to biodiversity; analyzing changes in the ecological niches of invasive species is of great significance for the prevention and control of biological invasions. Heilongjiang Province, which serves as a crucial ecological security barrier for China, has also faced invasions by invasive Asteraceae plants in recent years; however, the invasion patterns and ecological adaptation characteristics of these invasive plants remain unclear. Therefore, this study selected the representative Asteraceae species, *E. canadensis* and *E. annuus*, as the subjects of investigation. Based on data from the Heilongjiang invasive plant survey and supplementary field survey data, and in combination with distribution data from their native range in North America, the study employed methods such as ecological niche analysis and ecological niche overlap comparison and used Schoener’s D index. This study evaluates the potential suitable habitats for *E. canadensis* and *E. annuus* in Heilongjiang Province and analyzes the characteristics of niche shifts between their native and invaded habitats. The results indicate that both invasive plant species exhibit strong ecological conservatism; however, their ecological adaptation strategies in Heilongjiang Province differ, with *E. annuus* showing a higher degree of spread compared to *E. canadensis*.

## 1. Introduction

Biological invasion refers to the process by which non-native species invade a new habitat from their native range through natural or human-induced means. Invasive species pose a serious threat to biodiversity, agricultural, forestry, livestock, and fisheries production, as well as human health in the areas they invade [1,2,3]. With the advancement of globalization, the problem of invasive alien species has become increasingly severe, leading to global biodiversity loss and ecological degradation [4]. According to an assessment report by the Intergovernmental Science-Policy Platform on Biodiversity and Ecosystem Services (IPBES), invasive species account for approximately 60% of species extinctions and cause approximately $423 billion in economic losses each year [5,6]. Of the documented cases of animal and plant extinctions, approximately 60% are believed to be linked to invasive alien species. With the acceleration of global trade integration, global warming, and the rapid development of modern logistics and transportation systems, the chances of invasive species crossing geographic barriers and successfully establishing themselves have increased significantly. As one of the world’s most biologically diverse countries and a major hub for global trade and travel, China is facing an increasingly severe risk of biological invasions [7]. Particularly in areas where agricultural ecosystems and nature reserves overlap, invasive alien plants not only compete with native crops for resources such as light, water, and nutrients, but also disrupt the growth of native plants through allelopathy, posing a serious threat to national food security and ecological security [8]. Biological invasions occur on a wide scale; timely prediction of the distribution patterns of invasive alien species is the foundation for managing the risks posed by such species and an effective means of preventing their further spread.

Among the many groups of invasive plants, plants in the Asteraceae family have become one of the most successful invaders worldwide due to their extremely high reproductive capacity, diverse dispersal methods, and strong environmental adaptability. Among them, *E. canadensis* and *E. annuus* of the genus *Erigeron* are two highly representative invasive species [9]. Since their introduction to China, these two plants, native to North America, have successfully adapted to a wide range of latitudes and longitudes, extending from tropical and subtropical regions to cold-temperate zones, and have established stable populations in farmlands, orchards, along roadsides, on wasteland, and even at the edges of nature reserves. Abroad, *E. canadensis* has also long been regarded as a noxious agricultural weed. It can invade the soil of more than 40 types of crops, posing a threat not only to orchards and crop fields—such as those planted with corn, soybeans, and cotton—but also easily invading cultivated land, farms, and pastures. In China, *E. canadensis* has already caused damage to the country’s ecological environment and has also seriously affected the biodiversity of its ecosystems. The invasive plant *E. annuus* also secretes allelochemicals that inhibit the growth of surrounding plants, causing serious harm to agricultural ecosystems such as fall-sown crops, orchards, and tea plantations, and exhibits a strong ability to adapt to its environment. Studies have shown that in habitats at an elevation of approximately 1000 m, *E. annuus* exhibits high morphological plasticity, with functional traits such as specific leaf area adjusting in response to changes in environmental conditions. These traits are closely related to relative growth rate and resource acquisition capacity and form an important foundation for plants to maintain their ability to survive and reproduce under extreme environmental conditions such as low temperatures, drought, and short growing seasons [10,11]. In high-latitude cold-temperate regions, the ability of invasive plants to adapt to low temperatures, short growing seasons, and extreme climatic conditions is a key factor in their successful establishment and spread.

As a typical ecological security barrier in China characterized by a transitional continental monsoon climate between the cold and temperate zones, Heilongjiang Province’s unique climatic conditions and land-use patterns give the occurrence and spread of invasive plant species distinct regional characteristics and significant research value. In this regard, this study utilized data from the Heilongjiang Invasive Plant Survey to conduct field surveys in the Heilongjiang region, collect information on the ecological niche characteristics of species native to North America, and selected *E. canadensis* and *E. annuus* as the subjects of study. The study then employed spatial ecological niche analysis and comparisons of ecological niche overlap. This study systematically evaluated the potential suitable ranges of two invasive plant species and the changes in their ecological niches between their native and invasive ranges, revealing the environmental adaptation characteristics and potential spread risks of *E. canadensis* and *E. annuus*, and providing a theoretical basis for identifying risks posed by invasive plant species and monitoring key areas in Heilongjiang Province.

## 2. Materials and Methods

### 2.1. Acquisition and Screening of Distribution Data for E. canadensis and E. annuus

**Figure 1:**
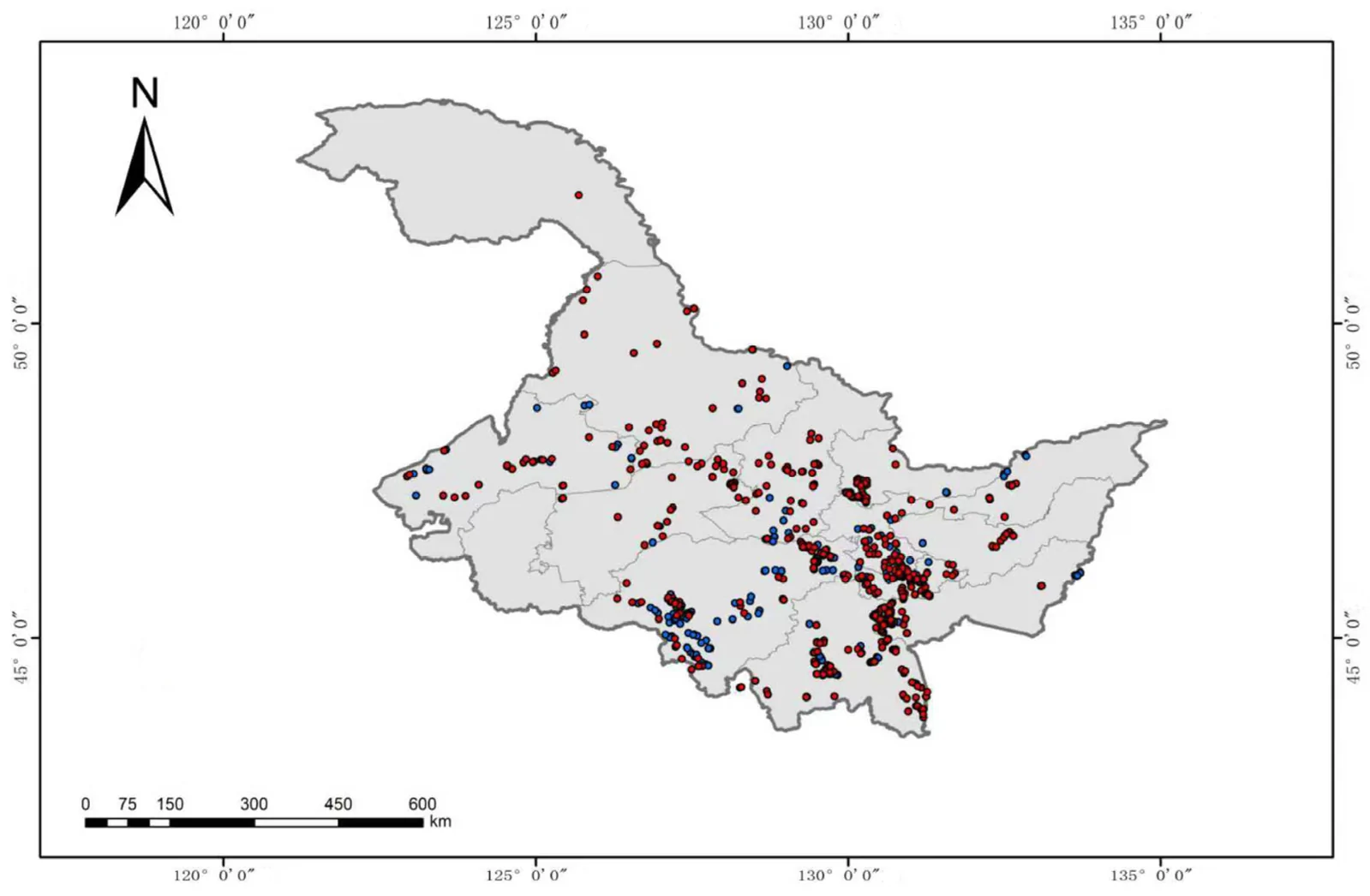
Geographic distribution points of *E. canadensis* and *E. annuus.* Red dots indicate *E. canadensis*, and blue dots indicate *E. annuus*. Longitude indicates east longitude, and latitude indicates north latitude.

Based on the “National Survey of Invasive Species in Forest, Grassland, and Wetland Ecosystems” project, this study obtained preliminary distribution data on invasive plants in Heilongjiang Province. Among the 30 invasive alien species identified in the survey, this study selected *E. canadensis* and *E. annuus*—the species with the widest distribution and the greatest ecological impact—as the primary subjects of investigation. At the same time, to fill gaps in the census data regarding forest edges and secondary habitats, our research team conducted targeted supplementary surveys from June to December 2023. A total of 700 additional transects were independently established along roadsides and in forest clearings, adding 1526 new field distribution points. Among these, 1080 were for *E. canadensis* and 446 for *E. annuus*. The geographic distribution of *E. canadensis* and *E. annuus* is shown in Figure 1. Within the study area, disturbed habitats primarily support communities dominated by herbaceous plants; common species include *Geum aleppicum* Jacq., *Agrimonia pilosa* Ledeb., and *Glycine soja* Siebold & Zucc., among others. Additionally, 412 reference specimens were collected. All locations were verified through GPS positioning and habitat imagery, resulting in a reliable species distribution dataset that provides a solid foundation for subsequent niche analysis and suitability area prediction.

To gain a comprehensive understanding of the global distribution pattern of the target species and to precisely define the characteristics of its native ecological niche in North America, this study integrated multi-source, heterogeneous database resources, including the Global Biodiversity Information Facility (https://www.gbif.org/zh/, accessed on 1 October 2026), the China Virtual Herbarium (https://www.cvh.ac.cn/, accessed on 1 October 2026), and the Plant Picture Bank of China (PPBC, https://ppbc.iplant.cn/, accessed on 1 October 2026). By setting “*Erigeron canadensis*” and “*Erigeron annuus*” as search keywords, we focused on extracting geographic distribution records containing complete latitude and longitude information, obtaining a total of more than 470,000 raw data entries, of which approximately 460,000 were for *E. canadensis* and approximately 10,000 were for *E. annuus*. To construct a reliable niche model, we first preprocessed the species distribution data by cleaning and organizing all collected locations, including removing duplicate records [12]. To further reduce spatial bias that may result from uneven sampling, we used ArcGIS 10.8 software to create a 1 km-radius buffer zone centered on each distribution point. If multiple cluster points existed within a buffer zone, only one was retained to ensure spatial independence among the points [11].

### 2.2. Acquisition of Climate and Environmental Variables

The current climate data required for this study were obtained from the WorldClim database (http://worldclim.org/, accessed on 1 October 2026), specifically the 19 bioclimatic variables (Bio1–Bio19) published therein. This dataset covered the period from 1970 to 2000, with a spatial resolution of approximately 1 km (30 arcseconds). Detailed descriptions of each environmental factor and a summary of the data sources were provided in Table 1.

### 2.3. Methods for Analyzing Niche Space and Overlap

#### 2.3.1. Background Point Construction and Environmental Variable Extraction

In analyses of niche stability and overlap, the number of background points and the definition of their spatial extent have a critical impact on the reliability of the results. Too few background points may lead to biased estimates of species habitat preferences, while too many will increase the computational burden and reduce the efficiency of the analysis [13,14]. To reasonably define the study area, this study was based on known distribution sites of *E. canadensis* and *E. annuus* in their native and invasive ranges. Using the R library (sf)( The R version used was 4.5.1), we constructed minimum convex polygons to define the accessible background areas for each species [15]. Within this range, 10,000 background points were randomly generated, and their corresponding environmental variables were extracted for subsequent niche-space and overlap analyses. Using a Geographic Information System platform, this study integrated the coordinate data of the distribution points of *E. canadensis* and *E. annuus* in their native and invasive ranges. By combining this data with a climate and environmental database, the study extracted environmental variable values for each point through spatial overlay analysis. The data was then formatted into a uniform format and saved as a .csv file, providing standardized input data for subsequent niche analysis and statistical modeling. This method had been widely applied in species distribution modeling and niche studies, and it effectively enabled the spatial matching of environmental information with species distribution data [16,17].

#### 2.3.2. Niche Quantification and Principal Component Analysis

This study adopted the ecological niche spatial analysis framework proposed by Broennimann [16,18] and, using the “ecospat” package in R, conducted a quantitative analysis of the ecological niche characteristics of *E. canadensis* and *E. annuus* in their native and invasive ranges. This method has enabled the systematic quantification of niche overlap, expansion, stability, and unfilled portions by projecting species distributions onto an environmental space constructed from principal components, thereby revealing dynamic changes in ecological niches during the species invasion process. Based on existing research, if an invasive species’ niche expansion index was less than 10%, the species was considered to exhibit strong niche conservatism during the invasion process. To eliminate multicollinearity among environmental variables and identify the dominant factors, this study employed the PCA-env method to perform dimensionality reduction on the seven key variables that were selected. By comparing the projected distributions of the species in their native and invasive habitats in a two-dimensional environmental space, we quantified the relative contribution of each factor to niche differences [19].

#### 2.3.3. Niche Overlap Indices and Significance Tests

We used Schoener’s D index and the I index based on Hellinger’s distance to quantitatively assess the degree of interspecific niche overlap [20]. Both indices had a range of 0–1; higher values indicated greater niche similarity, while lower values indicated greater niche differentiation [21]. This method was widely used in studies comparing the niches of invasive plants. To test the statistical significance of the relationship between the ecological niches of populations in their native range and those in the invaded range, we employed random-replacement-based tests for niche equivalence and niche similarity. In the past, equivalence tests determined whether two niches were statistically equivalent by constructing a null distribution of the D-value through random resampling of distribution points; if the observed D-value fell significantly outside the confidence interval (*p* < 0.05), the hypothesis that the two niches were completely equivalent was rejected [22]. Similarity tests were used to assess the degree of overlap between the ecological niches of different regions under given environmental conditions, thereby revealing characteristics of niche conservation or niche shift.

#### 2.3.4. Single-Factor Ecological Niche Analysis of Environmental Factors

Based on the spatial analysis of ecological niches, we further compared the niche occupancy of individual environmental factors between the native range and the invaded area. By visualizing the distribution characteristics of points from different regions and background points along the gradients of various climate variables, green and red were used to represent the coverage ranges of climate signals in native and invasive areas, respectively, while the solid green and red lines represented the climate density distributions of environmental background points in the two areas, respectively. This provided a clear illustration of the trends in how species adapt to key environmental factors during the invasion process [23].

## 3. Results

### 3.1. A Comparison of the Ecological Niches of E. canadensis in Invasive Areas and Native Habitats

#### 3.1.1. Spatial Characteristics of Climate Niche Based on Principal Component Analysis

**Figure 2:**
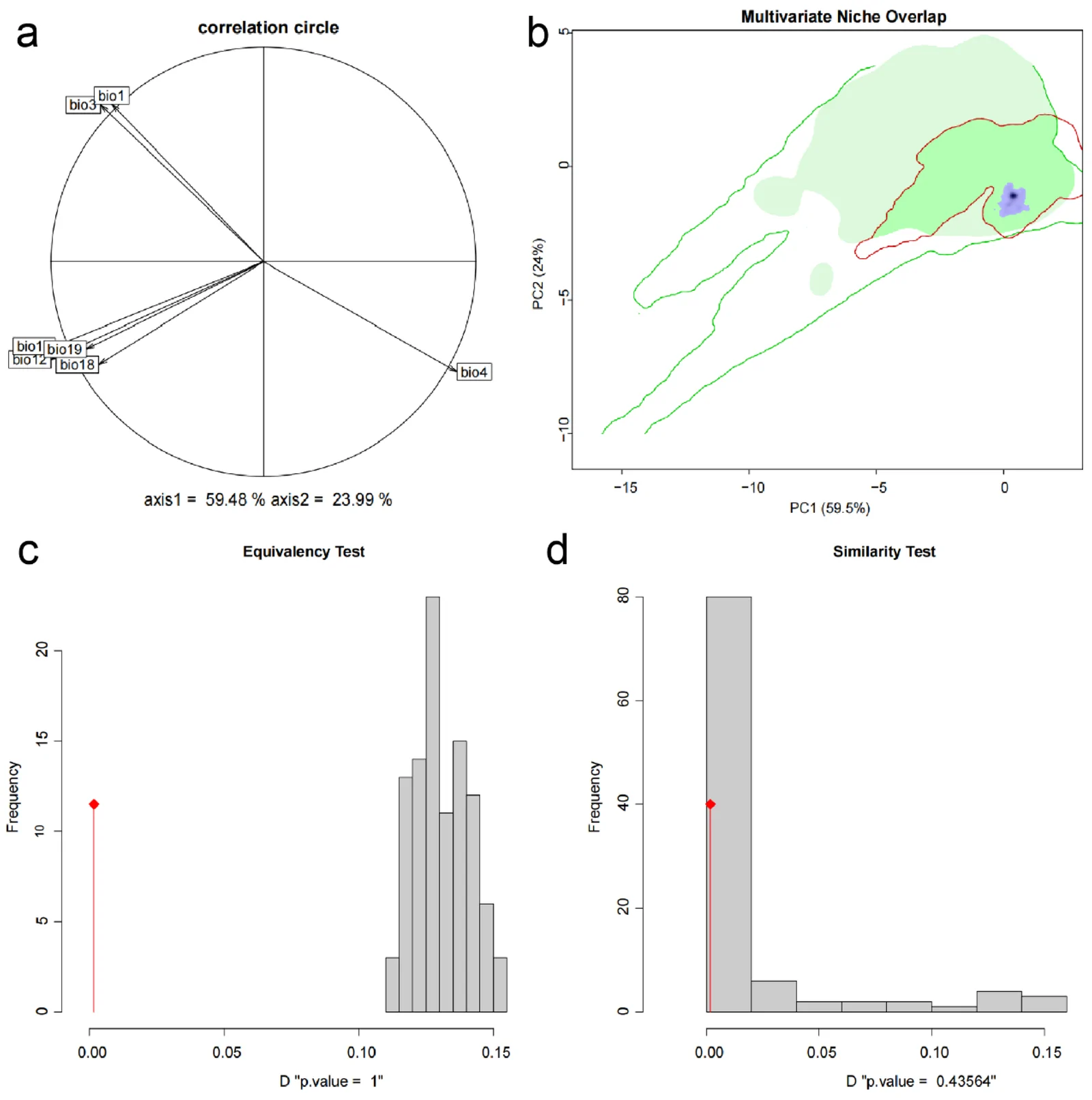
Comparison of climatic niche spaces and conservatism tests for *E. canadensis* in its native and invasive ranges. (**a**) The correlation circles for the two PCA axes illustrate the importance of environmental factors; (**b**) the native range (green) and invasive range (red)—the green area corresponds to the unfilled regions of the native niche, the purple area represents the overlap between the two niches, and the red area indicates the expansion of the invasive range; (**c**) Niche equivalence test—Comparison of the observed niche overlap frequency (D, red diamond-shaped line) with the simulated niche overlap (gray bars), 95% confidence interval, 100 iterations; (**d**) Niche similarity test.

Using principal component analysis (PCA), bioclimatic indicators for *E. canadensis* in its native range and invasive locations were projected onto a two-dimensional space; the results were shown in Figure 2. The results showed that the first two principal components of PCA explained 83.47% of the total environmental variation and effectively accounted for the first environmental gradient in species distribution. Principal Component 1 accounted for 59.48% of the variance and was primarily driven by environmental factors such as seasonal temperature variations, annual precipitation, precipitation during the wettest season, and precipitation during the warmest season. This indicated that the combination of water and heat in the environment was a key factor in the invasion of *E. canadensis*. Principal Component 2 accounted for 23.99% of the variance and was primarily influenced by environmental factors such as annual average temperature and isothermality, reflecting differences in thermal conditions.

#### 3.1.2. Analysis of Niche Overlap and Dynamic Indicators

Analysis of niche overlap based on Schoener’s D index indicated that niche overlap between the native range and the invasive range of *E. canadensis* was extremely low (D = 0.0017). This difference stemmed primarily from the significant geographical differences between the two regions, which resulted in the actual climatic habitats being highly spatially separated. The two-dimensional visualization results (Figure 2) clearly showed that the ecological niche of the invasive *E. canadensis* was almost entirely nested within the peripheral region of its native ecological niche, exhibiting distinct subset characteristics. Dynamic analysis based on the Stability, Expansion, Unfilling, and Exchange (COUE) framework further revealed the significant niche conservatism of *E. canadensis* (Table 2), with its stability index reaching the maximum value (Stability = 1.00). This indicated that the climatic resources in the invasive sites in Heilongjiang were fully within the existing range of adaptation of the species in its native range, and no niche shift had occurred. At the same time, the niche expansion index was zero (Expansion = 0.00), indicating that the species has not entered new climatic environments not found in its native range, but rather relies primarily on its original climatic survival strategies. It was worth noting that the unfilling index was extremely high (Unfilling = 0.95), suggesting that although there were a large number of suitable climatic habitats in Heilongjiang similar to those in its native range, only about 5% were currently occupied by *E. canadensis*. This “ecological niche vacancy” of as much as 95% indicated that the species still had significant potential for future spread and posed a risk of an outbreak. Schoener’s D value was low primarily due to significant differences in the environmental background between the species’ native range and the invaded area, rather than a substantive change in the species’ ecological niche. After correcting for environmental background, a Stability value of 1.00 indicated that the species had maintained a high degree of ecological niche conservatism during the invasion process.

#### 3.1.3. Statistical Tests for Niche Conservatism

The results of the niche equivalence and similarity tests statistically validated the divergence patterns (Figure 2); the equivalence test results were not significant (*p* > 0.05), indicating differences in the background environments of the two sites. However, no statistically significant differences were observed (*p* > 0.05), suggesting that, after adjusting for differences in the background environment, the niches in the two locations were statistically similar. Combined with the results from Stability = 1.0, the statistical tests strongly supported the niche conservatism hypothesis. This also indicated that the successful invasion of *E. canadensis* in Heilongjiang was primarily attributable to its precise utilization of its inherent broad ecological range, enabling it to find suitable habitats in the new environment.

#### 3.1.4. Unidimensional Niche Analysis of Key Environmental Factors

**Figure 3:**
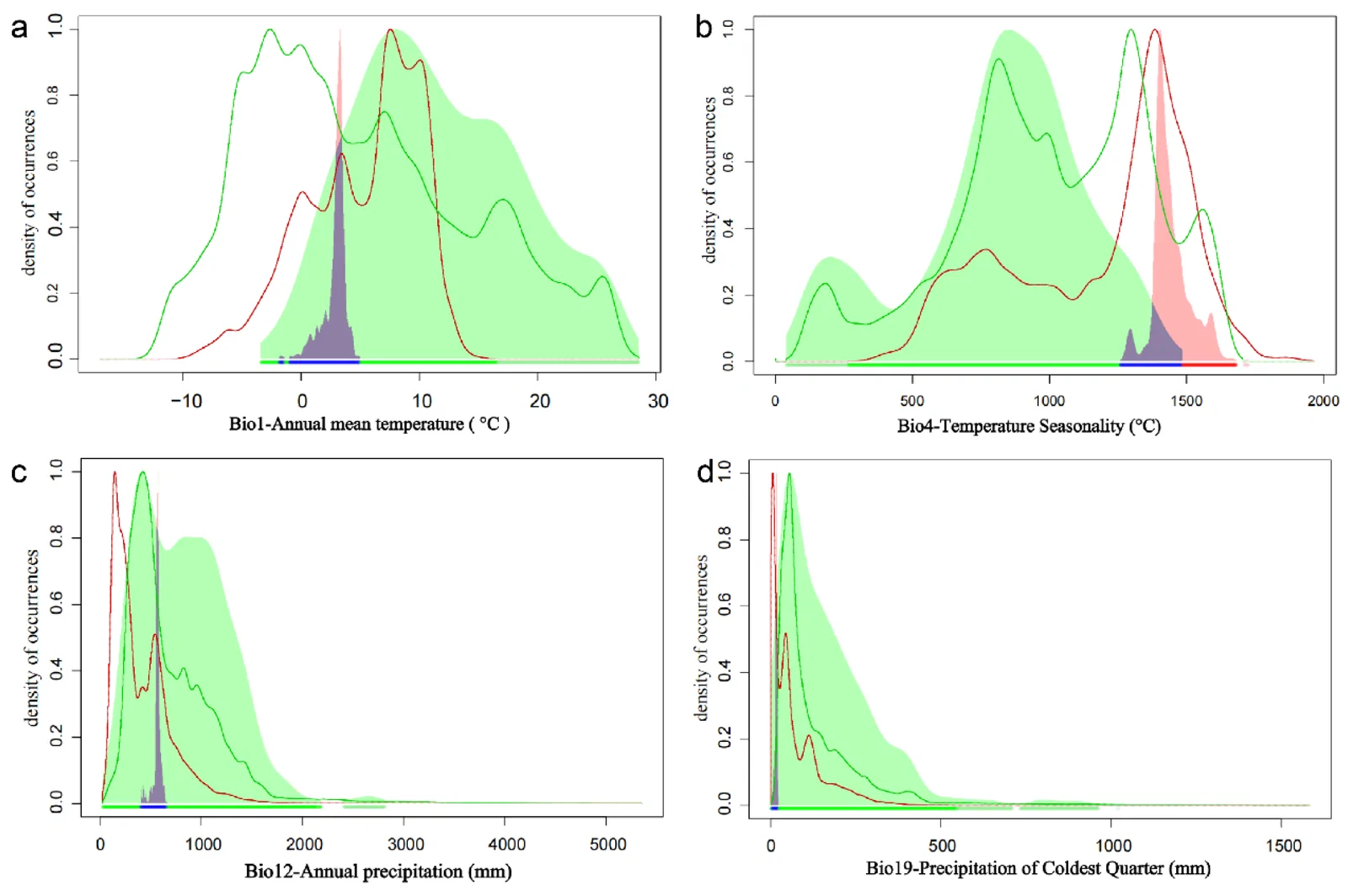
Climatic niche response curves of *E. canadensis*. (**a**–**d**) Climatic niche response curves for the four environmental factors. In each subfigure, the horizontal axis represents the range of variation in environmental factors, while the vertical axis represents changes in the distribution probability of *E. canadensis*; the blue shading indicates predicted niche overlap, the green shading indicates the predicted native niche, and the red shading indicates the predicted invasive niche; peaks in overlap indicate similar climate tolerance, while the width of the contours indicates the specificity of climate tolerance; the solid green and red lines represent 100% of the available environmental space in the native range and the invaded range, respectively.

To thoroughly analyze the response patterns of *E. canadensis* to key environmental factors, we selected the four dominant factors with the highest PCA contribution rates and plotted the niche density curve shown in Figure 3. The results showed that, for key environmental factors such as annual mean temperature and annual precipitation, the niche density curves for the invasive sites all fell entirely within the range of the niche curves for the native range; this was highly consistent with the conclusion of “Expansion = 0” from the multidimensional analysis. However, in terms of the distribution center of gravity, *E. canadensis* exhibited a clear adaptability to its environment, particularly with regard to seasonal temperature factors, as it was highly concentrated in the high-value range. This indicated that, during its invasion of Heilongjiang, *E. canadensis*—while not exceeding its physiological tolerance limits—prioritized habitats with large temperature fluctuations and distinct seasonal patterns for colonization, thereby completing its invasion and spread.

### 3.2. A Comparison of the Ecological Niches of E. annuus in Invasive Areas and Native Habitats

#### 3.2.1. Spatial Characteristics of Climate Niche Based on Principal Component Analysis

**Figure 4:**
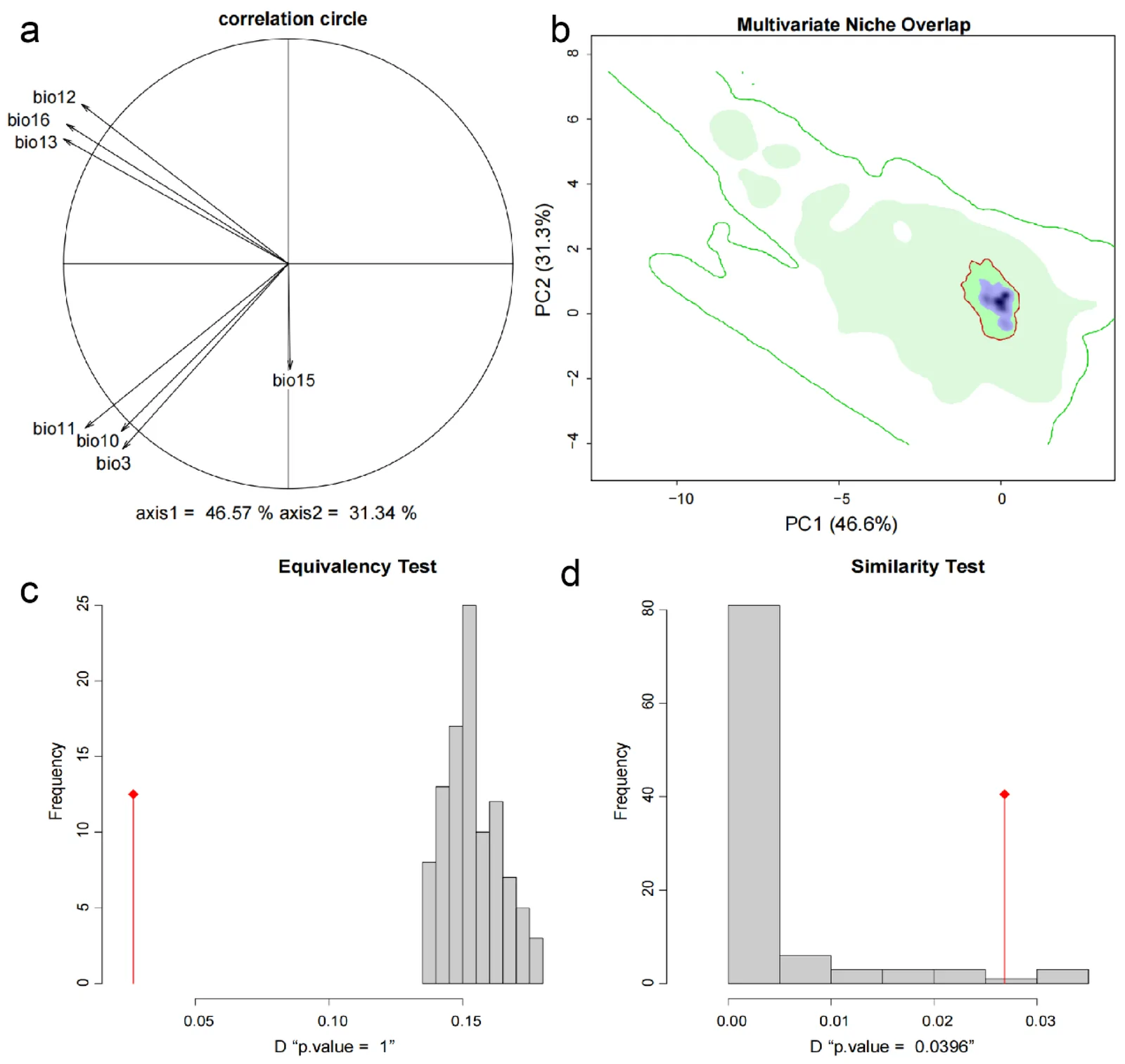
Comparison of climatic niche spaces and conservatism tests for *E. annuus* in its native and invasive ranges. (**a**) The correlation circles for the two PCA axes illustrate the importance of environmental factors; (**b**) the native range (green) and invasive range (red)—the green area corresponds to the unfilled regions of the native niche, the purple area represents the overlap between the two niches, and the red area indicates the expansion of the invasive range; (**c**) Niche equivalence test—Comparison of the observed niche overlap frequency (D, red diamond-shaped line) with the simulated niche overlap (gray bars), 95% confidence interval, 100 iterations; (**d**) Niche similarity test.

PCA was used to analyze the climatic niche distribution patterns of *E. annuus* in its native range and invasive areas (Figure 4). The results showed that the first two principal axes of PCA accounted for 77.91% of the variance and effectively captured the key environmental factors influencing the distribution of *E. annuus*. Principal component 1 accounted for 46.57% of the variance and was primarily driven by annual precipitation, precipitation during the wettest season, precipitation during the wettest month, and the average temperature during the coldest season, reflecting the abundance of moisture and basic thermal conditions in the environment. The second principal component accounted for 31.34% of the variance and was primarily influenced by the strong seasonality of precipitation, while also being associated with the mean temperature and isothermal conditions during the coldest season. The analysis revealed that the seasonal distribution pattern of precipitation and thermal conditions during extreme periods are the key environmental factors driving the spatial distribution differentiation of *E. annuus* in its native and invasive ranges.

#### 3.2.2. Analysis of Niche Overlap and Dynamic Indicators

Analysis based on Schoener’s D index showed that the niche overlap between the native and invaded sites for *E. annuus* was low (D = 0.026), indicating that the actual climatic habitats of the two sites were significantly spatially separated. However, this low degree of overlap did not indicate a change in the species’ ecology; as shown in Figure 4, the distribution of *E. annuus* in Heilongjiang was strictly limited by its original climatic adaptation boundary. Using the COUE framework to quantify niche dynamics (Table 3), the results revealed extreme niche conservatism. The stability index reached its peak (Stability = 1.00), and the niche expansion index was zero (Expansion = 0.00), exhibiting the same characteristics as *E. canadensis*. The high unfilling index (Unfilling = 0.515) revealed that, within the invasive environment, approximately 51.5% of the climatically suitable habitats similar to those in the native range have not yet been occupied by *E. annuus*. This indicated that the species was still in the middle stage of its invasion and expansion in Heilongjiang and still had significant potential for further spread in the future.

#### 3.2.3. Statistical Tests for Niche Conservatism

The results of the niche equivalence test (*p* > 0.05) and the niche similarity test (*p* < 0.05) further corroborated the aforementioned conclusions (Figure 4), indicating significant differences in the environmental backgrounds of the two sites. Similarity tests showed that, although environmental conditions in Heilongjiang differ from those in its native habitat, the *E. annuus* was not randomly distributed in their new environment. On the contrary, it exhibited a clear tendency toward ecological niche convergence, with the habitats it actually occupied showing a much higher degree of similarity than would be expected from random sampling. In other words, during its invasion, *E. annuus* tended to select habitats with climatic conditions similar to those of its native range, rather than adapting indiscriminately to all local environments.

#### 3.2.4. Unidimensional Niche Analysis of Key Environmental Factors

**Figure 5:**
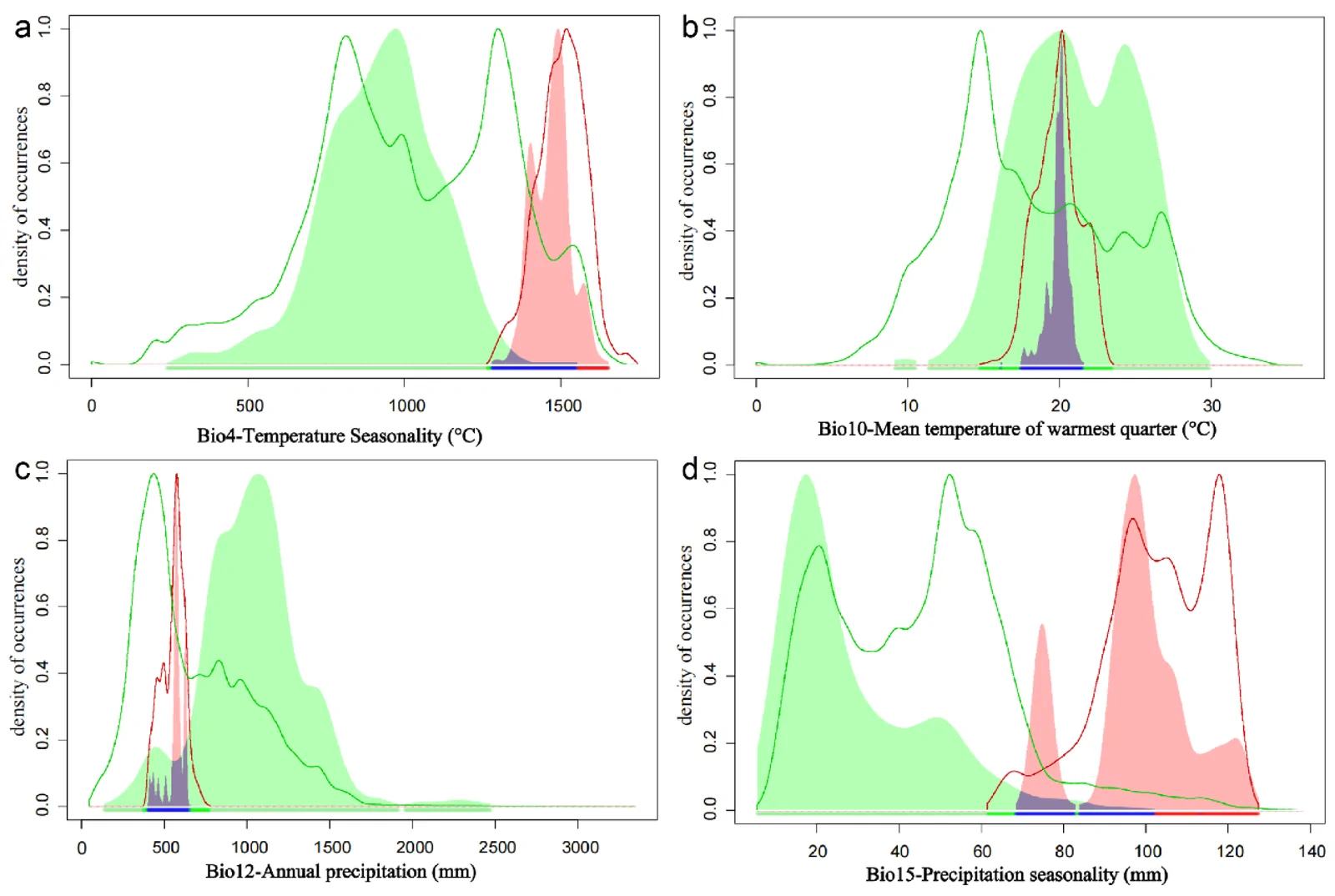
Climatic niche response curves of *E. annuus*. (**a**–**d**) Climatic niche response curves for the four environmental factors. In each subfigure, the horizontal axis represents the range of variation in environmental factors, while the vertical axis represents changes in the distribution probability of *E. annuus*; the blue shading indicates predicted niche overlap, the green shading indicates the predicted native niche, and the red shading indicates the predicted invasive niche; peaks in overlap indicate similar climate tolerance, while the width of the contours indicates the specificity of climate tolerance; the solid green and red lines represent 100% of the available environmental space in the native range and the invaded range, respectively.

To thoroughly analyze the response patterns of *E. annuus* to key environmental factors, we selected the four dominant factors with the highest PCA contribution rates and plotted one-dimensional niche density curves (Figure 5). The results showed strong consistency with the conclusions from the multidimensional analysis (where Expansion = 0) regarding key factors such as annual precipitation and the average temperature of the warmest season. However, in terms of distribution centers of gravity, there were distinct signs of environmental selection, particularly with regard to seasonal precipitation factors: invasive populations were highly concentrated in the high-value range, forming a stark contrast to the bimodal distribution of native populations. This suggested that, during its invasion of Heilongjiang, *E. annuus*—while not exceeding its physiological tolerance limits—prioritized colonizing habitats with significant seasonal variations in precipitation through environmental selection mechanisms, thereby adapting to the characteristics of the local temperate continental monsoon climate.

### 3.3. A Comparison of the Ecological Niches of E. canadensis and E. annuus

There are significant differences in the niche characteristics of the two plant species (Table 4). The niche overlap between *E. canadensis* and *E. annuus* is low; specifically, the D value for *E. canadensis* is only 0.0017, which is lower than the 0.0269 for *E. annuus*, indicating that the two plant species have a low degree of spatial niche overlap. Both plant species had a stability of 1.00 and an expansion of 0.00, indicating that both exhibited strong niche conservatism and did not show any significant niche expansion. The unfilled index for *E. canadensis* is 0.95, which is significantly higher than the 0.515 for *E. annuus*, indicating that there is a considerable gap between the current actual distribution of *E. canadensis* and its potential suitable range, whereas *E. annuus* makes relatively more full use of its potential ecological niche. The results of the similarity test showed that the *p*-value for *E. canadensis* was 0.435 (>0.05) and for *E. annuus* was 0.0396 (<0.05), indicating that there are also differences between the two species in terms of niche similarity. In addition, *E. canadensis* is primarily influenced by Bio4 (Temperature Seasonality) and Bio12 (Annual Precipitation), while *E. annuus* is primarily influenced by Bio10 (Mean Temperature of Warmest Quarter), Bio12, and Bio15 (Precipitation Seasonality), indicating that the two plant species exhibit different environmental adaptation characteristics in their niche occupation.

## 4. Discussion

### 4.1. Differences in the Invasion Stages, Potential Risks, and Environmental Adaptation Strategies of E. canadensis and E. annuus

*E. canadensis* and *E. annuus* exhibited marked niche conservatism during their invasion of Heilongjiang Province, meaning that the climatic range occupied by the invasive populations generally did not extend beyond that of their native range. This finding is consistent with the conclusions of recent studies, which indicate that most invasive plant species exhibit limited niche expansion during the invasion process, and their distribution is primarily constrained by their original climatic adaptability [24]. Further analysis revealed significant differences in the degree of niche unfilling between the two plant species. As shown in Table 4, *E. canadensis* exhibited a higher proportion of niche unfilling, while *E. annuus* exhibited a relatively lower proportion. This phenomenon indicates that *E. canadensis* has not yet fully occupied its potential range and remains in the early stages of expansion, whereas *E. annuus* has already established itself across a wide area. Previous studies have shown that the unfilled index is related to diffusion constraints and invasion time, indicating that species have not yet fully occupied all suitable habitats, although the results of this study support the niche conservation hypothesis. However, some studies have also found that invasive species may exhibit a certain degree of niche shift across different regions (e.g., plants of the genus *Rumex*), suggesting that niche dynamics are to some extent region-dependent [25,26,27]. Therefore, the results of this study are more likely to reflect the ecological characteristics of the two species during the current stage of their invasion in Heilongjiang Province rather than their long-term evolutionary trends.

*E. canadensis* and *E. annuus* exhibited marked niche conservatism (Table 4), but there were significant differences in the species’ responses to key environmental factors, reflecting different environmental adaptation strategies. Research findings indicate that the distribution of *E. canadensis* is primarily driven by seasonal temperature fluctuations and that it exhibits a high tolerance for temperature variations. This characteristic enables it to adapt to Heilongjiang’s typical temperate continental monsoon climate, thereby maintaining a stable distribution across a wide climatic gradient. Previous studies have shown that invasive plants with high temperature tolerance are generally able to adapt to a wider range of climatic fluctuations, thereby gaining a competitive advantage in high-latitude regions or areas with high environmental variability [28]. In contrast, *E. annuus* is more sensitive to seasonal variations in precipitation, with its distribution concentrated in areas characterized by a clear overlap between the rainy and hot seasons, indicating a stronger dependence on water and heat conditions. Although there is some overlap in the overall climatic conditions between the two species, differences in their selection of specific environmental conditions have, to some extent, reduced the intensity of direct interspecific competition. Relevant studies indicate that, in the process of adapting to new environments, invasive species often achieve niche differentiation through differential responses to various climatic factors in order to increase their chances of successful invasion [29].

### 4.2. Differences in Niche Selection Between E. canadensis and E. annuus

The results of the niche similarity test further revealed differences between the two species during the invasion process. The similarity test for *E. annuus* reached statistical significance (*p* < 0.05), while that for *E. canadensis* did not (*p* > 0.05). This suggests that *E. annuus* tends to select habitats in its invaded range that closely match the climatic conditions of its native range, exhibiting strong niche-selection characteristics. This colonization strategy, which is primarily driven by environmental matching, typically implies that species are highly dependent on specific environmental conditions, and their spatial distribution is largely constrained by the pattern of suitable habitats [30]. In comparison, *E. canadensis* generally exhibits characteristics of a conservative ecological niche; however, the similarity test did not reach statistical significance, which may indicate that its current distribution is still in the early stages of expansion and that it has not yet fully occupied all suitable habitats. This phenomenon is more common in the early stages of an invasion, when a species’ distribution is significantly constrained by dispersal limitations, resulting in a certain degree of “suboptimal occupation” [31]. In addition, the suitable habitat for *E. canadensis* is relatively widespread within the study area, which to some extent reduces its dependence on specific habitats and results in a relatively scattered distribution pattern. In summary, although *E. canadensis* and *E. annuus* exhibit strong niche conservatism, there are still significant differences in their ecological adaptation strategies in the cold-temperate region.

This may be related to its biological characteristics and the unique habitat conditions in Heilongjiang Province; the seeds of *E. canadensis* are small, numerous, and have a pappus, enabling them to be dispersed over long distances by the wind [32]. Given the strong spring winds in Heilongjiang and the widespread distribution of road corridors, the species is more likely to spread rapidly along traffic corridors and the edges of farmland. At the same time, *E. canadensis* exhibits strong tolerance to seasonal temperature fluctuations, enabling it to adapt to the significant annual temperature variations in the Heilongjiang region; as a result, it demonstrates a high potential for expansion and a large amount of unfilled ecological niche space. In contrast, *E. annuus* possesses facultative asexual reproduction, meaning it can still produce fertile seeds even under conditions of limited pollination [33,34]. This characteristic reduces its dependence on pollinators and helps it establish populations in high-latitude cold-temperate regions. Heilongjiang Province has a relatively short growing season and noticeably low temperatures in the spring, and *E. annuus* has increased its success rate of establishing itself in this new environment by improving the stability of its reproduction. In addition, *E. annuus* is more sensitive to seasonal variations in precipitation, and its suitable range is concentrated primarily in areas with favorable water and heat conditions, indicating that its spread is more significantly constrained by climatic conditions. The habitat heterogeneity of Heilongjiang Province—characterized by frequent agricultural activities, a dense road network, and pronounced freeze–thaw cycles—has also provided these two invasive plant species with a large number of suitable ecological niches for colonization. Among these factors, road disturbances and habitats along farmland edges may serve as important pathways for the rapid spread of *E. canadensis*, while moist, low-lying areas and regions subject to significant human disturbance are more conducive to the stable expansion of *E. annuus* populations. Both invasive plant species exhibited distinct niche conservatism in Heilongjiang Province, indicating that their invasion process was more aligned with the existing environment rather than achieved through significant niche expansion. However, there are clear differences between the two species in terms of their invasion processes: *E. canadensis* has not yet fully occupied its suitable habitat and still has significant room for expansion, whereas *E. annuus* has a relatively stable distribution within its suitable range and exhibits a higher degree of expansion, reflecting the different dispersal strategies adopted by the two species during the invasion process.

## 5. Conclusions

This study indicates that both *E. canadensis* and *E. annuus* exhibit significant niche conservatism in Heilongjiang Province, and the climatic space occupied by these invasive species generally does not extend beyond the range of their native habitats. However, there are significant differences between the two species in terms of niche selection, environmental adaptation, and invasion stages. *E. canadensis* exhibits a higher degree of unfilled habitat, and its distribution is primarily driven by seasonal temperature fluctuations, indicating that it is still in the expansion stage and has significant potential for further expansion; The *E. annuus* exhibits lower fill rates, significant niche similarity, and a stronger response to seasonal precipitation, indicating that it has already established a presence across a wide ecological range. Overall, although both plants exhibit strong niche conservatism, they have adopted different strategies for environmental adaptation and dispersal. Among them, *E. canadensis* poses a greater potential risk of dispersal, while *E. annuus* exhibits a higher current level of dispersal. The findings of this study can provide a scientific basis for risk assessment and targeted prevention and control of plants at different stages of invasion in Heilongjiang Province.

## Figures and Tables

**Table 1:** Climatic and environmental variables.

Bioclimatic Variables	Description	Units
Bio1	Annual Mean Temperature	°C
Bio2	Mean Diurnal Range	°C
Bio3	Isothermality	-
Bio4	Temperature Seasonality	-
Bio5	Max Temperature of Warmest Month	°C
Bio6	Min Temperature of Coldest Month	°C
Bio7	Temperature Annual Range	°C
Bio8	Mean Temperature of Wettest	°C
Bio9	Mean Temperature of Driest Quarter	°C
Bio10	Mean Temperature of Warmest Quarter	°C
Bio11	Mean Temperature of Coldest Quarter	°C
Bio12	Annual Precipitation	mm
Bio13	Precipitation of Wettest Month	mm
Bio14	Precipitation of Driest Month	mm
Bio15	Precipitation Seasonality	-
Bio16	Precipitation of Wettest Quarter	mm
Bio17	Precipitation of Driest Quarter	mm
Bio18	Precipitation of Warmest Quarter	mm
Bio19	Precipitation of Coldest Quarter	mm

Bio, Bioclimatic Variables.

**Table 2:** Indices of niche dynamic characteristics for *E. canadensis* in its native and invasive ranges.

Indices	Values	Significance
Schoener’s D	0.0017	Low overlap
Warren’s I	0.034	Low similarity
Stability	1.00	Highly stable
Unfilling	0.95	High invasion risk
Expansion	0.00	No expansion

**Table 3:** Indices of niche dynamic characteristics for *E. annuus* in its native and invasive ranges.

Indices	Values	Significance
Schoener’s D	0.026	Low overlap
Warren’s I	0.15	Low similarity
Stability	1.00	Highly stable
Unfilling	0.51	High invasion risk
Expansion	0.00	No expansion

**Table 4:** Comparison of ecological niches between *E. canadensis* and *E. annuus*.

Comparison Dimension	*E. canadensis*	*E. annuus*
Schoener’s D	0.0017	0.0269
Stability	1.00	1.00
Expansion	0.00	0.00
Unfilling	0.95	0.515
*p*	0.435	0.0396
Dominant factors	Bio4, Bio12	Bio15, Bio10, Bio12

## Data Availability

The original contributions presented in this study are included in the article. Further inquiries can be directed to the corresponding author.
